# Shared genetic mechanism between type 2 diabetes and COVID-19 using pathway-based association analysis

**DOI:** 10.3389/fgene.2022.1063519

**Published:** 2022-11-22

**Authors:** Kevin Chun Hei Wu, Qian He, Adam N. Bennett, Jie Li, Kei Hang Katie Chan

**Affiliations:** ^1^ Department of Biomedical Sciences, City University of Hong Kong, Hong Kong, Hong Kong SAR, China; ^2^ Jockey Club College of Veterinary Medicine and Life Sciences, City University of Hong Kong, Hong Kong, Hong Kong SAR, China; ^3^ Global Health Research Centre, Guangdong Provincial People’s Hospital, Guangdong Academy of Medical Sciences, Guangzhou, China; ^4^ Department of Electrical Engineering, City University of Hong Kong, Hong Kong, Hong Kong SAR, China; ^5^ Department of Epidemiology and Center for Global Cardiometabolic Health, Brown University, Providence, RI, United States

**Keywords:** COVID-19, type 2 diabetes, chemokine, immune system, pathway analysis, SARS- CoV-2, cytokine storm

## Abstract

Recent studies have shown that, compared with healthy individuals, patients with type 2 diabetes (T2D) suffer a higher severity and mortality of COVID-19. When infected with this retrovirus, patients with T2D are more likely to face severe complications from cytokine storms and be admitted to high-dependency or intensive care units. Some COVID-19 patients are known to suffer from various forms of acute respiratory distress syndrome and have a higher mortality risk due to extreme activation of inflammatory cascades. Using a conditional false discovery rate statistical framework, an independent genome-wide association study data on individuals presenting with T2D (N = 62,892) and COVID-19 (*N* = 38,984) were analysed. Genome-wide association study data from 2,343,084 participants were analysed and a significant positive genetic correlation between T2D and COVID-19 was observed (T2D: r for genetic = 0.1511, *p*-value = 0.01). Overall, 2 SNPs (rs505922 and rs3924604) shared in common between T2D and COVID-19 were identified. Functional analyses indicated that the overlapping loci annotated into the *ABO* and *NUS1* genes might be implicated in several key metabolic pathways. A pathway association analysis identified two common pathways within T2D and COVID-19 pathogenesis, including chemokines and their respective receptors. The gene identified from the pathway analysis (*CCR2*) was also found to be highly expressed in blood tissue *via* the GTEx database. To conclude, this study reveals that certain chemokines and their receptors, which are directly involved in the genesis of cytokine storms, may lead to exacerbated hyperinflammation in T2D patients infected by COVID-19.

## Introduction

Coronavirus disease 2019 (COVID-19) is caused by the severe acute respiratory syndrome coronavirus 2 (SARS-CoV-2). This virus mainly utilises the angiotensin-converting enzyme 2 (ACE2) receptor to enter human tissues. ACE2 is expressed within multiple human organs and is highly expressed in type 2 alveolar cells of the lungs, enabling the retrovirus to readily infect humans expressing a complicit receptor. Petrosillo et al. (2020) Though clinical symptoms of SARS-CoV-2 can vary in their severity, patients typically present with fever, headache, shortness of breath, and chest pain. Moreover, COVID-19 patients with comorbidities such as type 2 diabetes (T2D), hypertension, and cardiovascular disease exhibit a higher risk of severe complications and mortality compared to those without such associated comorbidities. Sanyaolu et al. (2020) Previous studies have suggested that regular inflammatory responses and immune system dysfunctions occur as a consequence of hyperglycaemia which, in turn, arises due to insulin resistance caused by hyperinsulinemia. Norouzi et al. (2021) Further, some studies also have shown that the more severe symptoms of COVID-19, including acute respiratory distress syndrome (ARDS) and respiratory failure, may be induced by an imbalanced immune response due to the over-production of cytokines (also known as a cytokine storm). Such imbalances can also increase vascular permeability and lead to multiple organ failures. Costela-Ruiz et al. (2020) In addition, the alveoli experience severe inflammatory reactions which initiate a dysfunctional cascade of inflammatory thrombosis in the pulmonary vasculature that can lead to local coagulopathies. (Abou-Ismail et al., 2020).

T2D is characterised as a condition of low-grade chronic systematic inflammation that can be measured in the form of elevated concentrations of the pro-inflammatory cytokines IL-1, IL-6, and tumour necrosis factor alpha [TNF-α]), as well as by levels of C-reactive protein and monocyte (macrophage) adhesion to the endothelium. King (2008) In addition, the concentrations of certain chemokines, including CCL1, CCL2, CCL4, and CXCL10, are significantly higher in patients with T2D. Patients with T2D also often face a higher risk of infection with diseases such as COVID-19 and show a poor prognosis and a higher risk of mortality. (Muller et al., 2005; Roncon et al., 2020). As such, studies have shown that the second most prevalent comorbidity in patients with severe COVID-19 infections is T2D. Roncon et al. (2020) Some studies have suggested that systemic inflammatory responses and immune system dysfunction might be related to the hyperglycaemia and insulin resistance caused by the dysfunction of beta cells in the pancreas. Norouzi et al. (2021) In addition, the occurrence of acute hyperglycaemia during a COVID-19 infection can significantly increase the concentrations of inflammatory mediators (cytokines and chemokines), thus enhancing the risk of multiple organ failure and acute cardiovascular events. (Norouzi et al., 2021).

Although some common physiological patterns have been observed between COVID-19 and T2D, the literature lacks any systematic analysis of the shared genetic loci between patients presenting the two conditions. This knowledge could help to develop better therapeutic strategies for COVID-19 patients with T2D symptoms. In this study, we analysed genome-wide association study (GWAS) summary statistics of T2D and COVID-19 using a pathway association-based approach and conditional false discovery rate (cFDR) to investigate the shared molecular pathways and genetic architectures between T2D and COVID-19. In addition, we used pleiotropy-based conditional and conjunctional FDR (conjFDR) statistics to discover common genetic determinants of the two traits.

## Materials and methods

### GWAS samples

GWAS summary statistics for T2D (https://www.ebi.ac.uk/gwas/studies/GCST006867) (Xue et al., 2018) and COVID-19 (https://www.ebi.ac.uk/gwas/studies/GCST011073) (COVID-19 Host Genetics Initiative, 2020) were obtained from the GWAS Catalog. The T2D summary statistics consisted of 62,892 case subjects and 596,424 control subjects. The COVID-19 summary statistics consisted of 38,984 case subjects and 1,644,784 control subjects. COVID-19 data were obtained from samples of European ancestry and T2D data were obtained from mixed samples of European (*N* = 655,666) and South Asian (*N* = 3,650) ancestry. The T2D data were generated by meta-analysing the Diabetes Genetics Replication and meta-analysis (34,940 cases and 114,981 controls), Genetic Epidemiology Research on Ageing (6,905 cases and 46,983 controls), and the full cohort release of the United Kingdom BioBank databases (21,147 cases and 434,460 controls) by using the software METAL. Willer et al. (2010) There were 5.1 million and 8.9 million genetic variants in the T2D and COVID-19 GWAS summary statistics, respectively. The lift over of single nucleotide polymorphisms (SNPs) and conversion of SNPs into rs IDs were performed using the NCBI Genome Remapping Service and UCSC Table Browser, respectively.

### Pleiotropy analyses

We used conditional quantile-quantile (Q-Q) plots (Andreassen et al., 2013), fold enrichment plots and linkage disequilibrium score regression (Bulik-Sullivan et al., 2015) (LDSC) to evaluate the pleiotropic enrichment and genetic correlations between T2D and COVID-19 GWAS summary statistics. We followed the instructions provided on the GitHub page (https://github.com/bulik/ldsc/) and performed the analysis using Python 3.

To improve the discovery rate of genetic variants correlated with T2D and COVID-19, we computed the cFDR statistics. Andreassen et al. (2013) The cFDR method is based on an empirical Bayesian statistical framework and used the GWAS summary statistics for a trait of interest alongside those for a conditional trait to estimate the posterior probability that an SNP has no association with the primary trait, provided that the *p*-values for that SNP in both the primary (T2D) and conditional (COVID-19) traits are as small as, or smaller than, the observed *p*-value. Thus, by re-ranking the test statistics of the main phenotype based on the strength of the connection with the secondary phenotype, this approach increases the likelihood of identification of genetic variations linked with the primary characteristic.

The conjFDR statistic was used to investigate the genetic variations shared by the two phenotypes. The conjFDR statistic is an extension of the cFDR statistic and is defined as the maximum of two mutual cFDRs for a specific SNP. It estimates the posterior probability that an SNP is null for either trait or both, given that the *p*-values for both phenotypes are as small as, or smaller than, the individual *p*-values for each trait. For cFDR and conjFDR, we chose a conservative threshold of 0.05 per pairwise comparison. Manhattan plots based on the conjFDR were created to highlight the genomic positions of the common genetic loci.

To minimise possible biases due to complicated linkage disequilibrium (LD) patterns, all analyses were performed after removing SNPs from the extended major histocompatibility complex (MHC) (hg19 position chromosome 6: 25,11,9106–33,85,4733) and the 8p23.1 (hg19 location chromosome 8: 72,42,715–12,48,3982) genomic regions.

We defined the independent significant SNPs according to the Functional Mapping and Annotation (FUMA) protocol (https://fuma.ctglab.nl/). SNPs having a conjFDR <0.05 and at *r* ^2^ < 0.6 with each other were considered independent significant SNPs and a fraction of the independent significant SNPS in approximate linkage disequilibrium with each other at *r* ^2^ < 0.1 were considered lead SNPs. In addition, we used the default parameters from FUMA to determine the distinct genomic loci and their borders.

### Genomic loci definition and functional annotation

We used SNPnexus (Oscanoa et al., 2020) to annotate the shared SNPs into genes and identify the overrepresented pathways for the genes nearest the identified shared loci between T2D and COVID-19.

### Gene-based analysis

PASCAL (Pathway scoring algorithm) (Lamparter et al., 2016) was applied to the T2D and COVID-19 GWAS summary statistics separately. Individual SNPs (*p* < 0.05) from the summary statistics were first mapped using a 20 kb window around the 5′ and 3’ UTRs. The maximum number of SNPs per gene allowed by PASCAL was 3,000. LD information was retrieved from the 1,000 Genomes European Panel and the significant *p*-value thresholds for T2D and COVID-19 were 2.26 × 10^–6^ (0.05/22,135 genes from the hg19 list) as the entire UCSC list (hg19) used by PASCAL to make calculations is included in this number of genes.

### Gene network analysis

FunCoup v.4.0 (https://funcoup4.scilifelab.se/search/) was employed to expand the list of significant genes between T2D and COVID-19 with their interactors. The FunCoup database combines ten different types of functional couplings among genes to infer functional association networks: protein interaction (PIN), mRNA co-expression (MEX), protein co-expression (PEX), genetic interaction profile similarity (GIN), shared transcription factor binding (TFB), co-miRNA regulation by shared miRNA targeting (MIR), subcellular co-localisation (SCL), domain interactions (DOM), phylogenetic profile similarity (PHP), and quantitative mass spectrometry (QMS). Gene networks for T2D and COVID-19 were constructed with five shared genes between T2D and COVID-19. Expansion parameters for constructing the gene networks included a confidence threshold (0.8), a maximum number of 30 nodes per expansion step, and a query depth of one. The network expansion approach was used to find the strongest interactors for each query gene while ignoring the ties between common neighbours. Furthermore, for each gene network established with the associated *p*-values, enriched term analyses (Kyoto Encyclopaedia of Genes and Genomes (KEGG), GO biological function, and GO molecular function) were conducted. After adjusting for multiple comparisons using false discovery rate (FDR), gene network representation depicts the most important KEGG pathways based on their q-values. The node sizes represent the gene relevance across the whole network, while each KEGG pathway’s involved nodes are denoted in black.

### Functional annotation

GENE2FUNC, one of the functions in FUMA (Functional Mapping and Annotation of Genome-Wide Association Studies) (https://fuma.ctglab.nl/), was used to annotate common genes between T2D and COVID-19 and their interactors. Several GENE2FUNC functions were utilised, including a heatmap of gene expression and an enrichment analysis of differentially expressed genes (DEG). Using GTEx v8 (54 tissue types) data, a gene expression heatmap was generated. On the related heatmaps, the average of normalised expression per label (zero means across samples) was presented. TPM (Transcripts Per Million) for GTEx v8 are the expression values. Heatmaps provide normalised expression values (zero mean normalisation of log2 transformed expression), with greater relative expression of a gene indicated by a deep red label and lower relative expression of a gene indicated by a dark blue label.

### Pathway enrichment analysis

**FIGURE 1:**
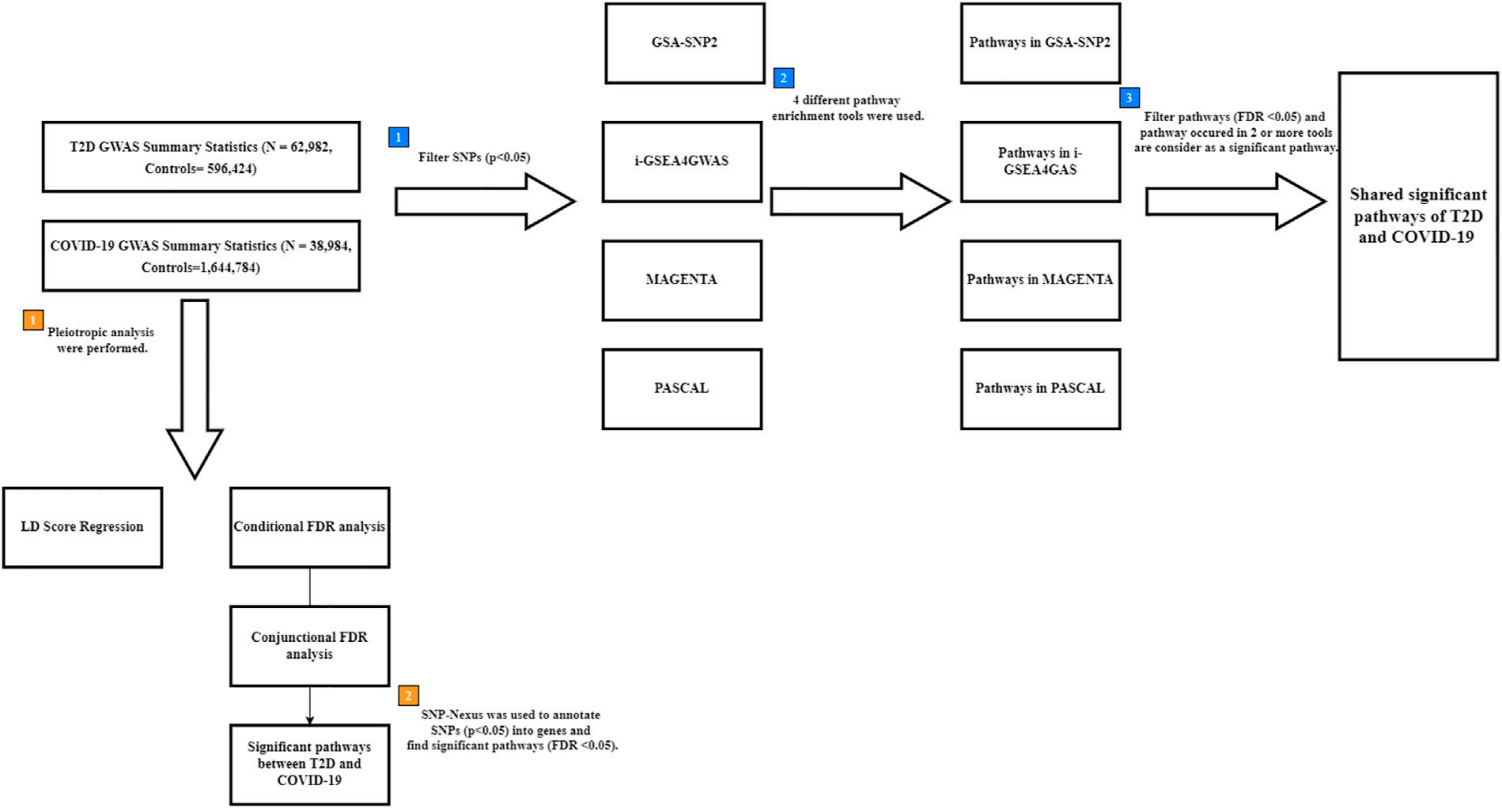
Flow chart for finding shared pathways between T2D and COVID-19. Blue square points describe the steps of multiple pathway enrichment analysis. Orange square points describe the steps of pleiotropic analysis. T2D: type 2 diabetes; COVID-19: coronavirus disease 2019; FDR: False Discovery Rate; LD: linkage disequilibrium; GWAS: genome-wide association study; SNPs: Single nucleotide polymorphisms.

To detect the pathways shared between T2D and COVID-19, four pathway enrichment analysis methods (GSA-SNP2 (Yoon et al., 2018), MAGENTA (Segrè et al., 2010), PASCAL (Lamparter et al., 2016), and i-GSEA4GWAS (Zhang et al., 2010)) were used to identify enriched pathways in each disease (Figure 1). GSA-SNP2 (Yoon et al., 2018) performs pathway-based analysis by testing the enrichment of associated genes in each pathway using Z-statistics of the random set models to assess pathways and a monotone cubic spline trend to determine SNP counts. MAGENTA (Segrè et al., 2010) performs pathway-based analysis on SNPs within a gene boundary using weighted Kolmogorov-Smirnov statistics that compare the ranks of genes within uniform distributions. PASCAL (Lamparter et al., 2016) calculates gene-based test statistics for all genes and carries forward the gene-based results to conduct a pathway-based test using chi-square statistics (including pathways with 10–200 genes only) that convert the corresponding *p*-value based on the pathway. The Benjamini–Hochberg procedure was used to account for multiple comparisons in the pathway-based analysis. i-GSEA4GWAS (Zhang et al., 2010) performs pathway analysis by using SNP label permutations to modify GWAS SNP *p*-values and rectify the genes and gene sets. It then multiplies the proportion ratio factor to the enrichment score to obtain the significant proportion-based enrichment score. Manhattan plots of the gene sets in each pathway were constructed and used to highlight the results of the association test for a given pathway.

Canonical pathways from curated gene sets (https://www.gsea-msigdb.org/gsea/msigdb/collections.jsp#C2) and ontology gene sets (https://www.gsea-msigdb.org/gsea/msigdb/collections.jsp#C5) in MSigDB (Subramanian et al., 2005; Liberzon et al., 2015) were used in the pathway enrichment analysis in all four pathway-based association approaches. Significant SNPs (*p* < 0.05) were mapped to genes if they were located within a range of 20 kb upstream or downstream of genes’ transcription start sites. In order to capture potential regulatory SNPs in a gene’s 5′ and 3′ untranslated regions and to prevent erroneous SNP-to-gene assignments brought on by wider windows, the 20-kb window offered the ideal width (Ghosh et al., 2013). Furthermore, we restricted the downstream analyses to pathways with 10–200 genes only in order to avoid testing over narrow or broad functional pathways (Wang et al., 2007) In addition, significant pathways were determined with a threshold of FDR <0.05.

### Material and data availability

The data underlying this article are available and downloaded in the GWAS Catalog (Buniello et al., 2019) at https://www.ebi.ac.uk/gwas/studies/GCST006867 (GWAS summary statistics for T2D) (Xue et al., 2018) and https://www.ebi.ac.uk/gwas/studies/GCST011073 (GWAS summary statistics for COVID-19) (COVID-19 Host Genetics Initiative, 2020) and can be accessed with GWAS Catalog study accessions GCST011073 and GCST006867 respectively.

## Results

### Pathway enrichment analysis of T2D

With respect to canonical pathways, we found 84 pathways to be the most significant (FDR <0.05) among the gene sets with a KEGG antigen processing and presentation pathway (*p*-value = 1.78 × 10^–13^, q-value = 3.45 × 10^–10^) using the GSA-SNP2 method (Supplementary Table S1A). In addition, among 198 pathways in total, the most significant pathway was the cyclin D associated events in the G1 pathway (Reactome) (*p*-value < 0.001, q-value = 0) with i-GSEA4GWAS (Supplementary Table S1B). Among 39 pathways, the most significant one was the maturity onset diabetes of the young (KEGG) (*p*-value = 7.78 × 10^–8^, q-value < 0.01) with PASCAL (Supplementary Table S1C). We also found the most significant pathway was the maturity onset diabetes of the young in the KEGG database (*p*-value = 5 × 10^–6^, q-value = 0) with MAGENTA (Supplementary Table S1D).

Using the gene ontology database, the most significant gene set in the Gene Ontology Biological Process category was the insulin secretion pathway out of 130 pathways in total (*p*-value = 1.61 × 10^–13^, q-value = 8.02 × 10^–10^) with GSA-SNP2 (Supplementary Table S1A). In addition, the most significant gene set in the Gene Ontology Biological Process category was the interferon-gamma-mediated signalling pathway out of 383 pathways in total (*p*-value < 0.001, q-value = 0) with i-GSEA4GWAS (Supplementary Table S1A). We found the most significant gene set in the Gene Ontology Biological Process category was the insulin secretion pathway among 112 pathways in total (*p*-value = 0, q-value = 0) with PASCAL (Supplementary Table S1C). We did not find any significant pathways among the gene sets with MAGENTA.

### Pathway enrichment analysis of COVID-19

In the canonical pathway database, we did not identify any significant pathways among the gene sets when using GSA-SNP2. However, we found 21 significant pathways among the gene sets, with Reactome chemokine receptors binding a chemokines pathway (*p*-value < 0.001, q-value = 0) with i-GSEA4GWAS (Supplementary Table S2A). Among the gene sets, 38 pathways were found to be significantly (FDR <0.05) associated with COVID-19 and the Reactome linked glycosylation pathway was the most significant (*p*-value = 0, q-value = 0) with PASCAL (Supplementary Table S2B). We did not find any significant pathways among the gene sets with MAGENTA.

With respect to gene ontology pathways, we did not find any significant pathways among the gene sets with GSA-SNP2. However, 23 pathways were significant among the gene sets, with the Gene Ontology Molecular Function G-protein coupled chemoattractant receptor activity pathway being the most significant (*p*-value < 0.001, q-value < 0.001), with i-GSEA4GWAS (Supplementary Table S2A). We found 151 pathways had a significant association with COVID-19 in the Gene Ontology Biological Process category, where the synapse assembly pathway was the most significant (*p*-value = 0, q-value = 0), with PASCAL (Supplementary Table S2A). We did not find any significant pathways among the gene sets when using MAGENTA.

### Common pathways between T2D and COVID-19

**FIGURE 2:**
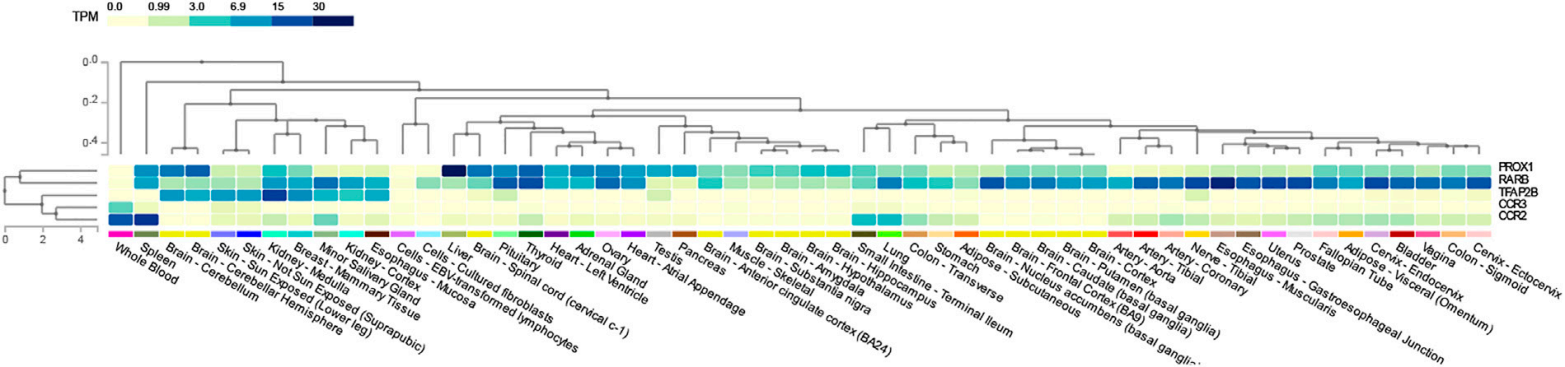
Shared genes tissue expression plot with GTEx v8 (54 tissues). Tissues are ordered by clusters for the plot. TPM: Transcript per million.

To determine the common pathways between T2D and COVID-19, we compared the significant pathways that were shared between them. We found four pathways (Supplementary Table S3A) in common between T2D and COVID-19 using iGSEA4GWAS: the chemokine binding, G-protein coupled chemoattractant receptor activity pathways (CCR2 and CCR3), the TFAP2 family pathway (TFAP2B), and the ventricular cardiac muscle cells differentiation pathway (RARB and PROX1) in the canonical pathway and gene ontology database. The chemokine binding pathway plays a role in recruiting immune cells to infection sites, and G-protein coupled chemoattractant receptor functions in mediating leukocytes’ chemotaxis and promoting innate and adaptive host immune responses. Furthermore, we found 15 pathways (Supplementary Table S3B) shared between T2D and COVID-19 using PASCAL, which were associated with various organs and biological processes, such as the heart, axons and calcium channels, in the canonical pathway and gene ontology database. However, no overlapping pathways were found between the four pathway-based analysis software. Five genes (*CCR2*, *CCR3*, *TFAP2B*, *RARB* and *PROX1*) (Figure 2) were used to construct the gene expression heatmaps with GTEx v8 (representing 54 tissues) to investigate their expression in all tissue types.

### Genetic correlation and genetic overlap between T2D and COVID-19

**FIGURE 3:**
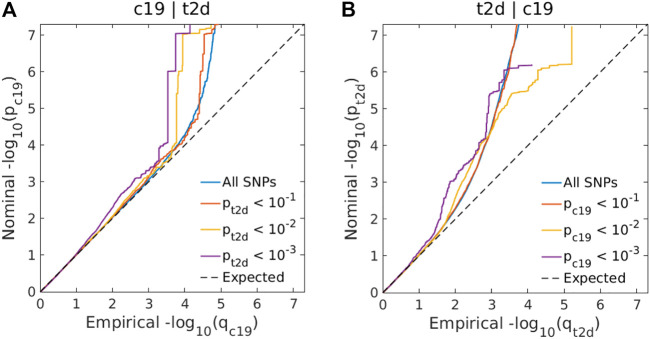
**(A)** Conditional quantile-quantile (Q–Q) plots of nominal vs. empirical coronavirus disease 2019 (COVID-19) –log_10_ *p*-values (corrected for inflation) below the standard genome-wide association study (GWAS) threshold of *p* < 5 × 10^–8^ as a function of the significance of the association with type 2 diabetes (T2D) at the levels of *p* ≤ 0.10, *p* ≤ 0.01, and *p* ≤ 0.001. **(B)** Conditional Q-Q plots of nominal vs. empirical T2D −log_10_ *p*-values (corrected for inflation) as a function of the significance of the association with COVID-19 at the level of *p* ≤ 0.10, *p* ≤ 0.01, and *p* ≤ 0.001. The dashed line indicates the null hypothesis. SNP: single-nucleotide polymorphism; c19: coronavirus disease 2019; t2d: Type 2 Diabetes.

**FIGURE 4:**
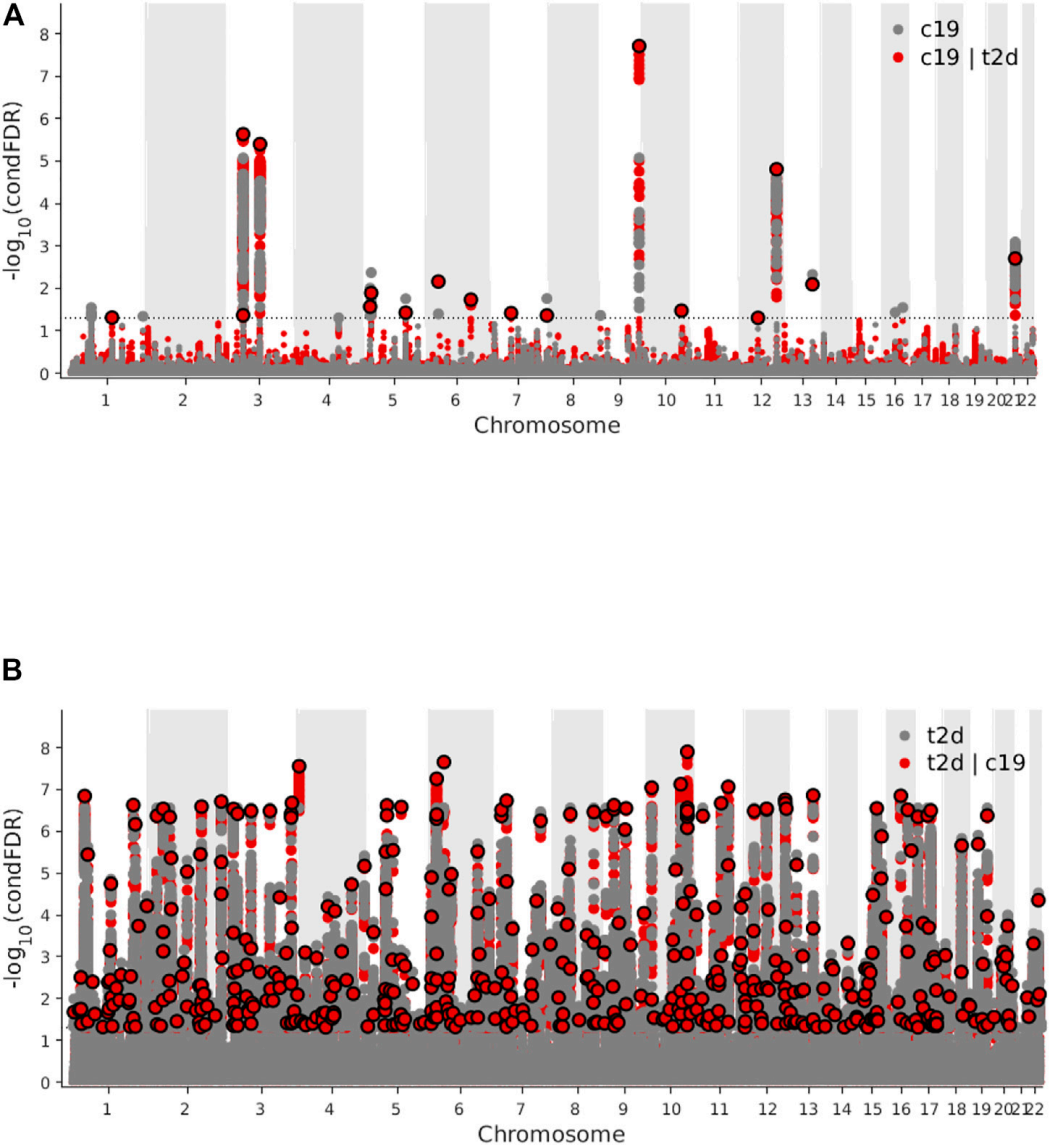
**(A)** Manhattan plot of type 2 diabetes (T2D) conditional upon coronavirus disease 2019 (COVID-19) for conditional false discovery rate (cFDR) < 0.05. **(B)** Manhattan plot of coronavirus disease 2019 (COVID-19) conditional upon type 2 diabetes (T2D) for conditional false discovery rate (cFDR) < 0.05.

Genome-wide LD score regression analyses showed significant positive genetic correlations between T2D and COVID-19 (r for genetic = 0.15, *p*-value = 0.01). We observed an enrichment of associations with COVID-19 across different levels of association with T2D (Figure 3A), indicating a small polygenic overlap between COVID-19 and T2D. We also constructed reverse conditional Q-Q plots (Figure 3B) for T2D conditional upon different levels of association with COVID-19. At a threshold of cFDR <0.05, we identified 471 loci associated with T2D conditional upon COVID-19 (Figure 4A). The reverse cFDR analysis revealed 17 loci associated with COVID-19 conditional upon T2D (Figure 4B).

### Functional annotation of shared loci between COVID-19 and T2D

**FIGURE 5:**
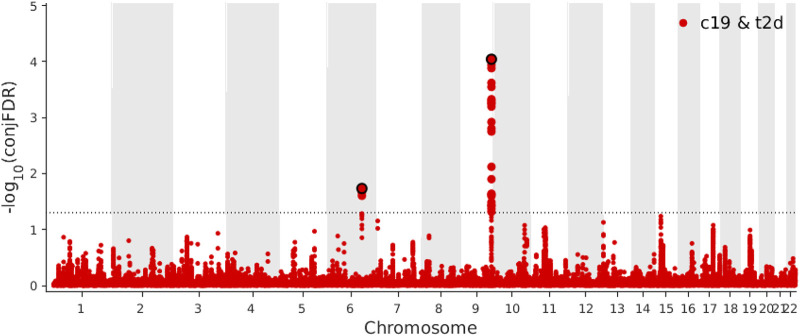
Manhattan plot of coronavirus disease 2019 (COVID-19) and type 2 diabetes (T2D) for conjunctional false discovery rate (conjFDR) < 0.05.

Based on a threshold of conjFDR <0.05, we identified two loci shared between COVID-19 and T2D: *ABO* (rs505922, intronic) and *NUS1* (rs3924604, intronic) (Figure 5). By comparing the effect directions of the shared independent loci (conjFDR <0.05), both these independent loci were showing consistent direction. One (rs505922) was showing positive effect while another one (rs3924604) was showing negative effect. These two genes were identified through two distinct SNPs using SNPnexus. (Oscanoa et al., 2020). We found eight pathways (Supplementary Table S4) to be significantly overrepresented among the genes nearest the identified loci shared between COVID-19 and T2D, with the defective DHDDs causing retinitis pigmentosa 59 pathway (*p*-value = 1.88 × 10^–4^) being the most significant.

### Gene-based analysis

PASCAL analysis revealed an association of 394 genes in T2D (Supplementary Table S5A) and an association of 58 genes in COVID-19 (Supplementary Table S5B). After comparing the significant genes in T2D and COVID-19, five genes (Supplementary Table S5C) were found to be shared between T2D and COVID-19: *PTPRD, CSMD1, MAGI1, ASIC2, DAB1*.

### Gene network analysis

**FIGURE 6:**
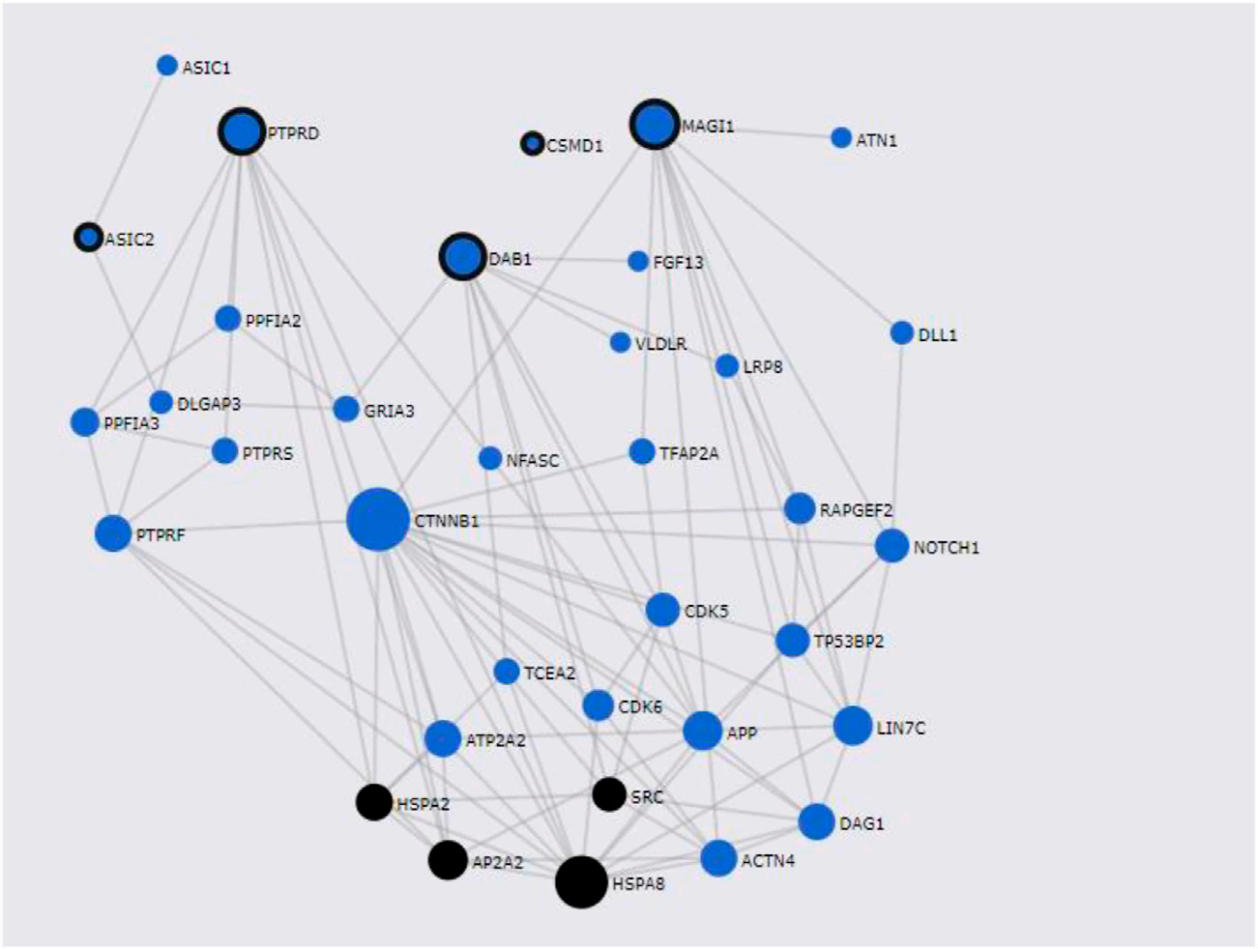
Shared gene networks constructed with Pascal associated genes and its FunCoup interactors, Node sizes scale to emphasize gene importance in the whole network, while participating nodes for KEGG metabolic endocytosis pathway are marked in black. Nodes encircled in black are shared genes between COVID-19 and T2D.

FunCoup has detected several interactors for T2D and COVID associated genes identified by PASCAL. Gene network for shared genes between T2D and COVID-19 was constructed (Figure 6) after including 35 genes (30 subnetwork genes plus 5 query genes) (Supplementary Table S6A) and considering 92 links between them. Enrichment analysis for KEGG and GO terms (Supplementary Table S6B) has shown association of different biological processes, including endocytosis (q-value = 4.58 × 10^–3^), central nervous system development (q-value = 3.64 × 10^–9^), and protein domain specific binding (q-value = 5 × 10^–9^) etc.

### Functional annotation

**FIGURE 7:**
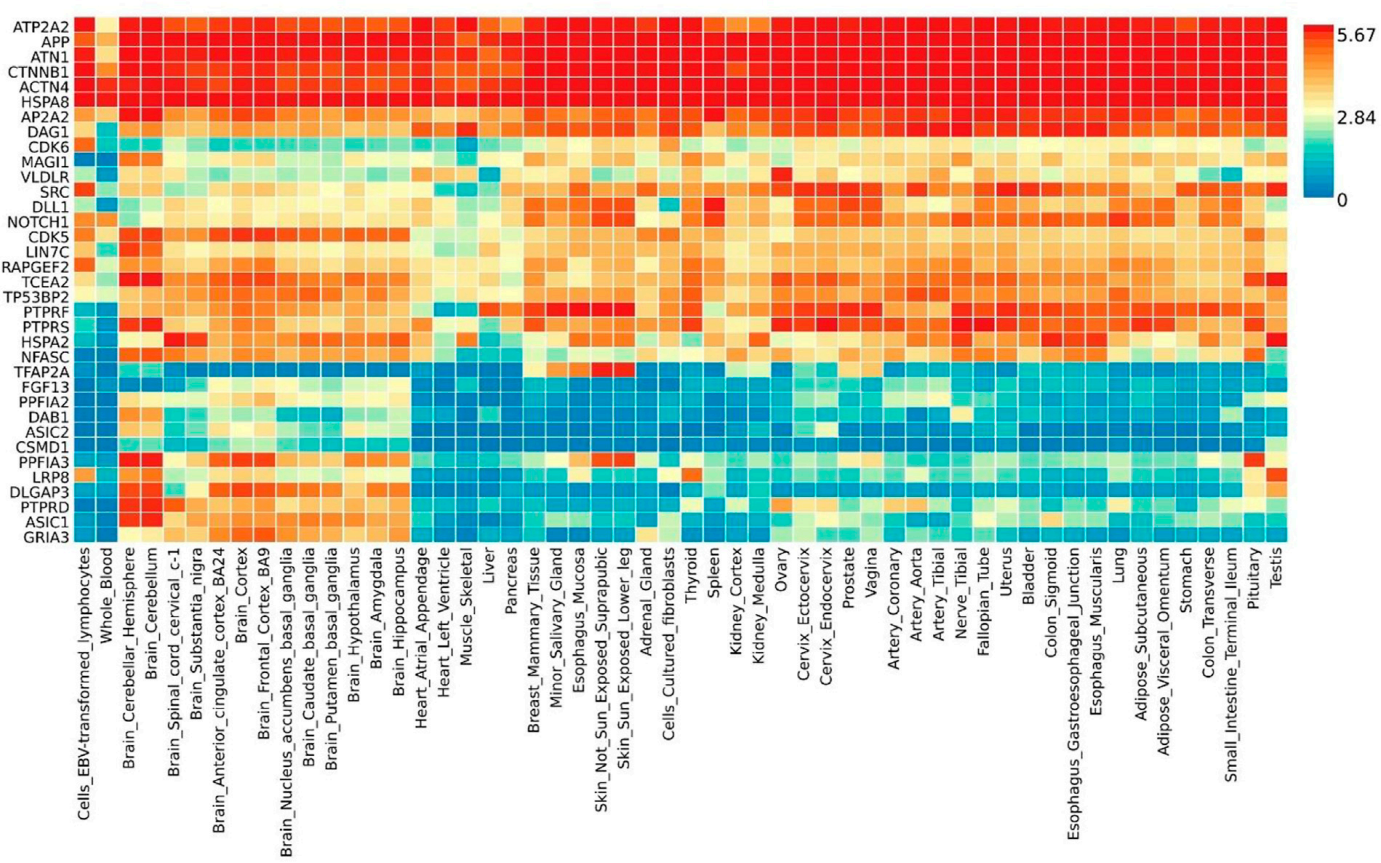
Shared gene expression heatmaps constructed with GTEx v8 (54 tissues) Gene and Tissues are ordered by clusters for the heatmap.

**FIGURE 8:**
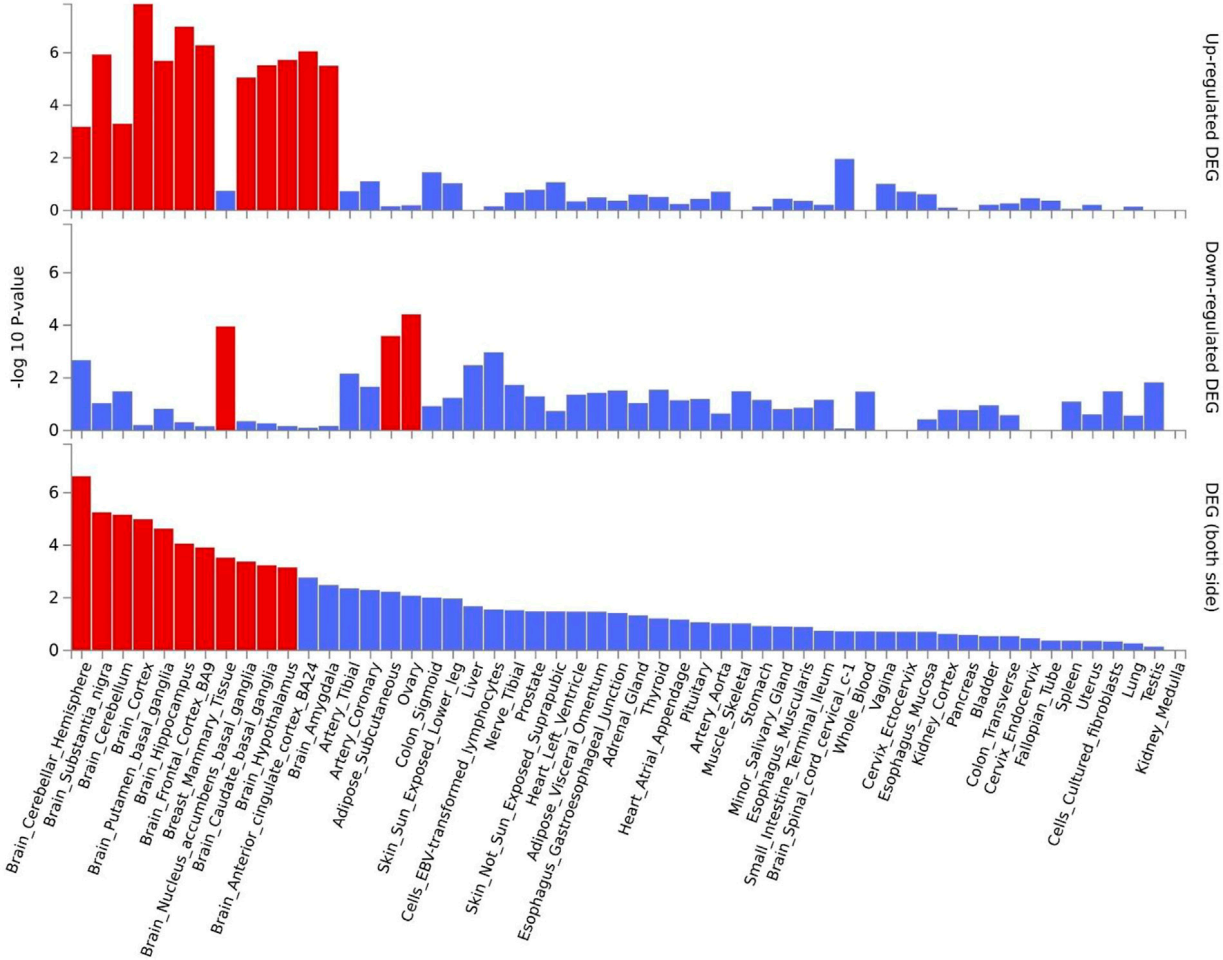
Shared genes DEG plots constructed with GTEx v8 (54 tissues). Significantly enriched DEG sets (P_bon_ < 0.05) are highlighted in red.

Gene expression heatmap based on GTEx V8 RNA-Seq data for T2D and COVID-19 associated genes togethers with FunCoup interactors (5 genes +30 subnetwork query genes) (Figure 7) was constructed. Specifically, six genes (*ATP2A2*, *APP*, *ATN1*, *CTNNB1*, *ACTN4* and *HSPA8*) display increased expression levels in all available tissues compared with other genes included in the analysis. Moreover, most of the genes display the trend showing low or moderate relative expression levels on brain tissues. Differential expression gene analysis (DEG) (Figure 8) showed that all brain tissues were highly upregulated while breast mammary tissue, ovary tissue and adipose tissue were highly downregulated contrast to other tissues.

## Discussion

In this study, the shared genetic association between T2D and COVID-19 was investigated using four pathway enrichment analysis methods (GSA-SNP2, i-GSEA4GWAS, PASCAL and MAGENTA) in Gene Ontology and canonical pathway databases. After the correction for multiple comparisons using FDR ≤0.05 as the criterion for a significant pathway, both PASCAL and i-GSEA4GWAS identified significant pathways between T2D and COVID-19 (15 pathways and 4 pathways respectively). The top shared pathways between T2D and COVID-19 included the chemokine binding, G-protein coupled chemoattractant receptor activity pathways and the ventricular cardiac muscle cellular differentiation pathway. Among these shared pathways, CCR2 and CCR3 are the common chemokines receptors found between the molecular mechanisms. Further, in our gene-based analysis, five genes (*DAB1*, *ASIC2*, *MAGI1*, *CSMD1* and *PTPRD*) were shared between T2D and COVID-19. DEG analysis showed that these genes and their interactors mainly have a low or moderate expression level in the cerebellar hemisphere and cerebellum tissue. We also identified two shared SNPs (rs505922 and rs3924604) with conjFDR <0.05.

Among the shared genes between T2D and COVID-19 by PASCAL, the *PTPRD* gene encodes a member of the protein tyrosine phosphatase family which regulates cellular processes including differentiation and cell growth. *DNMT1* promotes the DNA methylation of *PTPRD*, thus contributing to the silencing of insulin signalling in T2D patients. Chen et al. (2015) *PTPRD* expression levels were lower and correlated in T2D. A previous study showed that SARS-CoV-2 interacts with *open reading frame 8* (*ORF8*) and *ORF8* interacts with *DNMT1.* Gordon et al. (2020) There might be a possible interaction for *PTPRD* between T2D and COVID-19. Furthermore, the expression pattern of the identified gene *CSMD1* in COVID-19 shows important similarities to basal cell-related carcinomas. Iwabuchi et al. (2022) While *CSMD1* might be associated with insulin sensitivity and lipid levels in type 2 diabetes patients, this gene may also have an important impact on long-term elevated HbA1c levels. (Goswami et al., 2016).

Our study provides insight into the links between T2D and COVID-19 in terms of susceptibility loci, genes and genetic pathways. We used pathway association and statistical analyses to evaluate the association between T2D and COVID-19. We found that T2D was related to COVID-19 but not *vice versa*, which is consistent with the current findings in the literature. There was also a lack of evidence for T2D associated with the risk of COVID-19. Au Yeung et al. (2021) Previous studies have revealed that COVID-19 patients can develop new-onset diabetes and severe metabolic complications of pre-existing diabetes due to high concentrations of glycated haemoglobin. Accili (2021) Another study has also proposed a causal relationship regarding the onset of type 1 diabetes due to the observation that an increase in the risk of developing type 1 diabetes appears to coincide with COVID-19 infection status. Unsworth et al. (2020) Significant pathways were shared between the two phenotypes in the pathway association analysis, including the chemokine binding and the chemokine receptors pathways. Even though the GWAS Summary Statistics for T2D were predominantly from European descent, which might affect the results of the genetic correlation between T2D and COVID-19. We still got similar results comparing to the study. Chang et al. (2021) We found two SNPs that were shared between T2D and COVID-19 (conjFDR <0.05). Further, our analysis revealed a significant positive correlation between the genetic variants associated with T2D and COVID-19. The conjFDR analysis of the two shared genes, *ABO* and *NUS1*, indicated that COVID-19 and T2D might be connected to immune function and chemokine activation status. Certain glycosyltransferases are related to immune cell recruitment such as leukocyte rolling through binding to selectins. Sperandio et al. (2009) Protein glycosylation is also associated with the regulation of T-cell activation. (Marth and Grewal, 2008).

The *ABO* gene potentially plays a role in the pathogenesis of COVID-19 and T2D. This gene encodes a glycosyltransferase that catalyses the transfer of nucleotide donor sugars to the H antigen to form A and B antigens. Patenaude et al. (2002) These transferase enzymes and nucleotide donor sugars also induce the production of inflammatory mediators such as IL-6 and TNF-α in the endothelium. Rizzo et al. (2014) A study hypothesised that *ABO* might be a severity locus in COVID-19 rather than an infection locus. Goel et al. (2021) A previous study also mentioned that blood type O might have a lower risk of SARS-CoV-2 due to the anti-A or anti-B antibodies contribute to viral neutralization or anti-A isoagglutinins bounded by SARS-CoV-2 blocked the interaction between the virus and ACE2 receptor. Arend (2021) In addition, a study also shown that people with the O blood type were having a lower risk of developing T2D. Fagherazzi et al. (2015) However, the underlying molecular mechanisms under that is still unclear. A previous study has also shown that *ABO* is a possible marker for T2D and COVID-19 as people with blood type A are at a higher risk of T2D and being infected with SARS-CoV-2 simultaneously, whereas blood type O may be associated with a lower risk of SARS-CoV-2 and T2D. (Meo et al., 2016; Li et al., 2020).

The *NUS1* gene, located at chromosome 6q22.1, is involved in dolichol synthesis and protein glycosylation. It encodes a membrane protein-Nogo-B receptor (NgBR), which is a subunit of cis-prenyltransferase. NgBR is an enzymatic complex that is essential for protein N-glycosylation, a process that can alter the structure and function of proteins by steric influences or by mediating interactions with glycan-binding proteins. (Harrison et al., 2011). Pro-inflammatory cytokines can also change the cell surface N-glycosylation of endothelial cells, indicating that glycosylation can modulate inflammatory vascular diseases. (Reily et al., 2019). In addition, Nogo-B interacts with Interferon-induced transmembrane protein 3 (IFITM3) and IFITM3 suppressed the SARS-CoV-2 for the induce of IL-6 production. (Clement et al., 2022). Furthermore, a previous study has shown in mouse models that N-glycosylation defects, such as those in which N-acetylglucosaminyltransferase-IVa is inactive, can impair insulin release and lead to hyperglycaemia by abnormal N-glycosylation of pancreatic beta-cell glucose transporter-2 (GLUT-2) in T2D. (Ohtsubo, 2010; Reily et al., 2019). Another study shown that NGBR knockout mice resulted in increased blood glucose, insulin resistance and beta-cell loss. (Chen et al., 2021).

Multiple pathway enrichment approaches were used to identify pathways that are shared between the two diseases. Both pathways identified by our analysis—the chemokine binding pathway and G-protein coupled chemoattractant receptor activity pathway—were directly linked to immune-related activities through chemokines. Chemokines can bind to G-protein coupled seven-transmembrane receptors (chemokine receptors) on the surfaces of leukocytes, to glycosaminoglycans attached to the core proteins of cell surfaces and proteoglycans in the extracellular matrix. Kuschert et al. (1999) Glycosaminoglycans activate chemokines, triggering them to mobilise and recruit various immune cells. Chemokine receptors belong to the G protein coupled receptor superfamily and recruit dendritic cells. Murdoch and Finn (2000) The findings from our pathway analysis implicate that certain chemokine receptors are common between T2D and COVID-19, namely, CCR2 and CCR3. Previous studies have shown that some chemokine receptors, including CCR2, are involved in the pathogenesis of COVID-19 and of T2D. (Coperchini et al., 2020; Lim et al., 2021).

CCR2 is a chemokine receptor for various monocyte chemoattractant proteins (MCPs), such as CCL2, CCL7 and CCL8, and is a key functional receptor for CCL2. CCL2, also known as monocyte chemoattractant protein 1 (MCP-1), is a chemokine that binds to CCR2 and CCR4. CCL2 attracts monocytes, memory T-cells, and dendritic cells to sites of infection or inflammatory areas triggered by tissue damage. Daly and Rollins (2003) CCL2 also shows chemotactic activity for monocytes and basophils. Glycosaminoglycan binding and oligomerisation are essential for CCL2 to exert its *in vivo* effects and mediate the cytokine storm inflammatory response. Proudfoot et al. (2003) CCL2 is a key factor in the pathology of cytokine storms and promotes monocyte recruitment by acting both locally and remotely. The expression of CCL2 by insulin-producing cells can lead to insulitis and islet destruction. Also, CCL2 concentrations are higher in the plasma of T2D and COVID-19 patients than in the plasma of healthy controls. (Kretowski et al., 2016; Huang et al., 2020).

CCR3 binds and reacts to a variety of chemokines such as CCL5 and CCL7. CCL5 act as a chemoattractant for blood monocytes, memory T-helper cells and eosinophils. Baggiolini and Dahinden (1994) A study showed that CCL5 was upregulated in COVID-19 patients compared to non-COVID-19 patients. Zhou Y. et al. (2020) Furthermore, patients with T2D were significantly higher in CCL5 levels as compared to the control group. Herder et al. (2006) CCL7 attracts macrophages during inflammation and is found at elevated levels in bronchoalveolar lavage fluid (BALF) from severe COVID-19 patients. Zhou Z. et al., 2020

Further, we observed no overlap among the pathways/gene sets identified by PASCAL and i-GSEA4GWAS in COVID-19. As each analytical method adopts a different statistical procedure with various underlying assumptions, including the chi-square test statistic (PASCAL) and Kolmogorov-Smirnov-like statistics (i-GSEA4GWAS), to compute the gene scores and pathway scores, different analytical methods may identify different top-ranking pathways. Shared pathways or genes of T2D and COVID-19 identified by different methods can be further validated using experiments or various publicly available databases to provide support for the findings. We used GTEx gene expression data to validate the genes of the chemokine and chemokine receptor pathways as delineated in the gene ontology database (*CCR2* and *CCR3*). We found that these two genes exhibit a moderate level of expression in whole blood tissue. It is reasonable for us to speculate that the upregulation of the chemokine receptors related to immune response might contribute to a cytokine storm.

Our study has several limitations. Firstly, it is challenging to investigate the commonly shared molecular mechanisms between traits that experience constant mutational changes such as COVID-19. Further studies which utilise animal models are required to determine the causal genetic variants or genes that underlie the shared associations detected in this study and deduce whether the same causal genetic variants are involved in COVID-19 and T2D.Secondly, we could not identify another replication or validation cohort because we employed all the GWAS summary statistics to maximise the statistical power of both phenotypes. Lastly, our analysis was mainly based on data derived from the European population, so the results may not be applicable to different ethnic backgrounds. Despite these limitations, this is one of the first studies that examine the genetic overlap between COVID-19 and T2D using a comprehensive genetic analysis augmented with a pathway-based association analysis. Our study revealed little or no overlap between T2D and COVID-19 among European individuals. The novel loci and the shared pathways implicated that immunity is key to commonly shared molecular mechanisms between COVID-19 and T2D. The pathway association analysis provided significant support for the importance of chemokines and their receptors in T2D and COVID-19 aetiology.

In conclusion, our study has demonstrated genetic pleiotropy between T2D and COVID-19 and has identified shared genetic loci (*ABO* and *NUS1*) which were validated with a pathway-based analysis. Our results suggest a complex interplay of immune system-related gene pathways in the pathophysiology of chemokines and chemokine receptors. These findings are important for the development of actionable targets for novel therapies to treat COVID-19 patients with T2D and provide important implications for COVID-19 genetic aetiology.

## Data Availability

The original contributions presented in the study are included in the article/Supplementary material, further inquiries can be directed to the corresponding author.
